# Thirst and blood pressure regulation in a novel angiotensin II receptor type 1a knockout rat

**DOI:** 10.14814/phy2.71042

**Published:** 2026-08-07

**Authors:** Caroline B. Ferreira, Sean D. Stocker

**Affiliations:** ^1^ Department of Neurobiology University of Pittsburgh School of Medicine Pittsburgh Pennsylvania USA

**Keywords:** angiotensin II, angiotensin II receptor, blood pressure, hypotension, thirst

## Abstract

Angiotensin II (AngII) plays a pivotal role in body fluid balance through AngII type 1 receptors (Agtr1) expressed in circumventricular organs to stimulate thirst. Blockade of central Agtr1 attenuates thirst to hypotension but not acute hypernatremia. Data in genetic knockout models are limited as systemic AngII does not stimulate thirst in mice, and Agtr1a^−/−^ mice are severely hypotensive. Therefore, we generated a novel Agtr1a^−/−^ rat using CRISPR‐Cas9 to produce an 11‐bp frame‐shift deletion in exon 3. Baseline mean arterial blood pressure (ABP) was approximately 10 mmHg lower in Agtr1a^−/−^ vs. wild‐type (WT) rats with no differences in heart rate. Depressor responses to ganglionic blockade, vasopressin receptor blockade, or angiotensin converting enzyme inhibition were not different between strains. Systemic AngII (40 ng/min, IV) significantly increased water intake and mean ABP in WT rats, but these responses were absent in Agtr1a^−/−^ rats. Hypotension‐induced thirst produced by the vasodilator diazoxide (25 mg/kg, IV) stimulated water intake in WT but not in Agtr1a^−/−^ rats. In contrast, water intake stimulated by hypernatremia (2 M NaCl, IV) or overnight water deprivation was not different between strains. These findings support a critical role for Agtr1a in thirst induced by AngII and hypotension but not by hypernatremia or overnight water deprivation.

## Graphical abstract

Regulation of arterial blood pressure (ABP) is largely preserved in angiotensin II receptor 1a knock‐out (Agtr1a^−/−^) rats. However, deletion of Agtr1a attenuates thirst in response to AngII and hypotension but not intracellular dehydration.

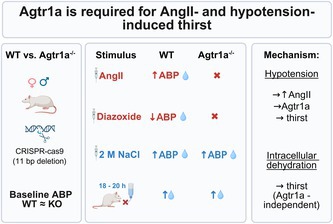

## 1 INTRODUCTION

The renin‐angiotensin system (RAS) plays a pivotal role in the regulation of body fluid homeostasis by coordinating cardiovascular, renal, and behavioral responses to fluid imbalance. Activation of the RAS occurs primarily during extracellular fluid volume depletion, such as hypotension and/or hypovolemia, which stimulates renin release and production of angiotensin II (AngII) (Fitzsimons, 1969; Leenen & Stricker, 1974; Stricker et al., 1979). AngII acts peripherally to promote vasoconstriction and sodium retention but also centrally to increase sympathetic nerve activity, alter neuroendocrine function, and stimulate fluid intake. In regard to the latter, thirst responses to extracellular fluid volume depletion depend on AngII as nephrectomy or inhibition of angiotensin‐converting enzyme (ACE) markedly attenuates the ingestion of water induced by hypotension and hypovolemia (Evered & Robinson, 1981; Houpt & Epstein, 1971; Johnson et al., 1981; Katovich et al., 1979; Stocker et al., 2000).

The central actions of AngII are largely mediated by angiotensin II type 1 receptors (Agtr1) (Fitzsimons, 1998). Intracerebroventricular administration of Agtr1 antagonists, such as losartan, markedly reduces pressor and dipsogenic responses to central AngII administration in rats (Kirby et al., 1992; Simpson et al., 1978; Widdop et al., 1994). In addition, thirst responses to central AngII are attenuated in Agtr1a^−/−^ (Li et al., 2003) or Agtr1b^−/−^ (Davisson et al., 2000) mice. Consistent with these observations, intracerebroventricular administration of Agtr1 antagonists attenuates water intake stimulated by hypotension or hypovolemia but not to acute hypernatremia (Buggy & Jonklaas, 1984; Fregly & Rowland, 1992; Simpson et al., 1978). Parallel studies using knockout models are limited. Agtr1a^−/−^ mice display reduced water intake in response to hypovolemia but not to water deprivation (Matsuda et al., 2017). Such experiments in Agtr1a^−/−^ mice are potentially confounded by two factors. First, systemic AngII does not reliably stimulate thirst in mice (Kobayashi et al., 1979; Rowland et al., 2003; Rowland & Fregly, 1988). Second, Agtr1a^−/−^ mice are profoundly hypotensive with baseline ABP approximately 20 mmHg lower than wild‐type controls (Chen et al., 2005; Ito et al., 1995; Sugaya et al., 1995). This is a potentially major confounding variable since the level of ABP influences thirst responses to several dipsogenic stimuli (Evered, 1992; Robinson & Evered, 1987; Stocker et al., 2001; Stocker et al., 2002).

To overcome these limitations, we generated a novel Agtr1a^−/−^ rat using CRISPR/Cas9 and 11‐bp frameshift deletion in exon 3. Importantly, baseline ABP was approximately 10 mmHg lower in the Agtr1a^−/−^ versus wild‐type rats but no differences in heart rate. The Agtr1a^−/−^ was targeted as this is the predominant isoform expressed in circumventricular organs and the paraventricular nucleus of the hypothalamus–key regions involved in fluid balance and cardiovascular control (Lenkei et al., 1997; Lenkei et al., 1998). Using this novel Agtr1a^−/−^ rat, we assessed fluid intake and arterial blood pressure (ABP) in response to systemic AngII, hypotension, acute hypernatremia, and water deprivation.

## 2 MATERIALS AND METHODS

### 2.1 Animals

All experimental procedures were approved by the Institutional Animal Care and Use Committee at the University of Pittsburgh (Protocol# 24085562) and followed the National Institutes of Health Guide for the Care and Use of Laboratory Animals. Agtr1a^−/−^ Sprague Dawley rats were generated at the Medical College of Wisconsin by pronuclear injection of a CRISPR‐Cas9 ribonucleoprotein complex targeting the sequence AGCGGTATAGACTGCCCACA into one cell Crl:SD (Charles River Laboratories) rat embryos, as previously described (Fehrenbach et al., 2021; Mehrotra et al., 2020; Spires et al., 2018). Founder animals were genotyped via PCR using the following primers: forward 5′‐CGTCTTTCTTCTCAATCTCGCCT‐3′; reverse 5′‐TGATGCAGGTGACTTTGGCC‐3′; followed by fragment analysis. Separate mutant strains were generated by backcrossing mutant founders to the parental Crl:SD strain. SD‐Agtr1a^em7Mcwi^ strain was established harboring an 11‐bp frame‐shift deletion in exon 3 (chr17:34,176,425–34,176,435 (rn7)). The colony is maintained by breeding heterozygous animals (Agtr1a^+/−^) to produce wild‐type (WT) and knockout (Agtr1a^−/−^) littermates for experiments. An ear punch sample was collected and used for PCR genotyping prior to weaning and results were blinded to the experimenter. Rats were housed in a temperature‐controlled room (22°C ± 1°C) with a 12‐h light–dark cycle (lights on 7 am–7 pm). Standard chow (Prolab® IsoPro® RMH 3000) and tap water were available ad libitum, except where noted.

### 2.2 General procedures

Male and female Agtr1a^−/−^ and WT littermates (12–14 weeks of age) were anesthetized with isoflurane (1.5%–2% in 100% O_2_) and instrumented with femoral arterial (Instech Laboratories; BTPE‐50) and venous (Braintree Sci.; #9049‐12‐00) catheters. The depth of anesthesia was confirmed by the absence of a withdrawal reflex to hind paw pinch. The catheters were exteriorized subcutaneously between the scapulae and secured to a vascular access button (Instech Laboratories; cat#VABR2B/22) or an infusion harness (Harvard Apparatus) protected by a steel swivel spring. Arterial and venous catheters were filled with 500 U/mL heparinized saline and flushed daily with 40 U/mL heparinized saline. Rats were treated with Ethiqa (0.65 mg/kg, SC) and enrofloxacin (2 mg/kg SC) and singly housed in metabolic cages (Techniplast). Water intake was recorded every 24 h. Experiments began 4–5 days later. On the day of the experiment, the animals were transported to the laboratory in the home metabolic cage and then permitted to acclimate to the experimental room for a minimum of 2 h. Animals with a vascular access button were connected to a swivel (Instech Laboratories; cat#KVABR2T25), and food was removed from the cages. All experiments were conducted between 9 am and 3 pm.

### 2.3 Experiment 1: Baseline hemodynamics

An initial set of experiments tested the extent by which deletion of the Agtr1a altered baseline hemodynamics and the regulation of ABP. After a 2‐h acclimation period, arterial lines were connected to DTX‐1 transducer and BPM‐832 Dual Pressure Monitor (CWE, Inc). ABP signals were digitized (1 kHz) using a Micro1401 and Spike2 Software (Cambridge Electronic Design). Heart rate was derived from the ABP signal. Baseline ABP and heart rate were recorded for 1 h. Then, a series of injections were performed to assess the contribution of various pressor systems to baseline ABP. The vasopressin 1a receptor antagonist [β‐Mercapto‐β,β‐cyclopentamethylenepropionyl^1^, O‐me‐Tyr^2^, Arg^8^]‐Vasopressin (10 ug/kg, IV; Sigma‐Aldrich #V2255) (Stocker et al., 2022) was administered. Approximately 1 h later, the ganglionic blocker hexamethonium (30 mg/kg, IV; Sigma‐Aldrich # H0879) (Stocker & Sullivan, 2023) was given. After a 3 h washout and return to baseline values, the angiotensin‐converting enzyme inhibitor captopril (3 mg/kg, IV; Sigma‐Aldrich # C4042) was administered (Evered & Robinson, 1984). Data was averaged into 10 s bins. Peak changes (10 s) within 10 min after injection were compared to baseline values (300 s).

### 2.4 Experiment 2: Effect of Agtr1a deletion on thirst stimulated by AngII, hypotension, hypernatremia or water deprivation

Male and female WT and Agtr1a^−/−^ littermates were instrumented with femoral arterial and venous catheters at least 4 days before experiments. On the day of the experiment, rats were transported to the laboratory and allowed to acclimate as described above. Food was removed, and the water bottle was replaced with a lickometer to measure both cumulative water intakes and latency to drink (Kinsman et al., 2020). Any animal that ingested >0.5 mL before the experiment began was not tested that day. To test whether Agtr1a deletion eliminated dipsogenic and pressor responses to AngII, an initial set of experiments infused angiotensin II (40 ng/min, 2.5 mL over 60 min, IV; Sigma‐Aldrich # A9525) (Fitzsimons, 1998; Kinsman et al., 2020). Next, thirst and hemodynamic responses were measured in response to the following paradigms: (1) hypotension produced by bolus injection of the vasodilator diazoxide (25 mg/kg, IV; Sigma‐Aldrich # D9035) (Stocker et al., 2000), (2) IV infusion of 2 M NaCl (2.5 mL over 30 min), and (3) 18–20 h water deprivation. Cumulative water intake (±0.5 mL) and urine output (±0.5 mL) were measured every 15 min over 60 min throughout all experiments. One challenge was tested per day, tests were performed every other day, and each animal was studied under all paradigms unless excluded due to catheter loss.

At the end of experiments, a subset of rats was anesthetized with isoflurane (1.5%–2% in 100% O_2_) and 500uL of blood was collected for analysis of plasma electrolytes using an EasyElectrolyte Analyzer (Medica). In addition, the heart and kidneys were harvested and weighed.

### 2.5 Data analysis

Data are presented as mean ± SD. The number of animals and sex are indicated in each figure. Individual data points are plotted when possible. Statistical comparisons were performed using ANOVA or *t*‐tests, as appropriate based on the number of groups and comparisons. When a significant *F*‐value was obtained, Bonferroni or unpaired *t*‐tests were used for post hoc analysis. Chi‐square test analyzed the proportion of rats that drank within each genotype. A *p*‐value ≤ 0.05 was considered statistically significant.

## 3 RESULTS

### 3.1 Experiment 1: Characteristics, baseline hemodynamics, and regulation of ABP

**FIGURE 1:**
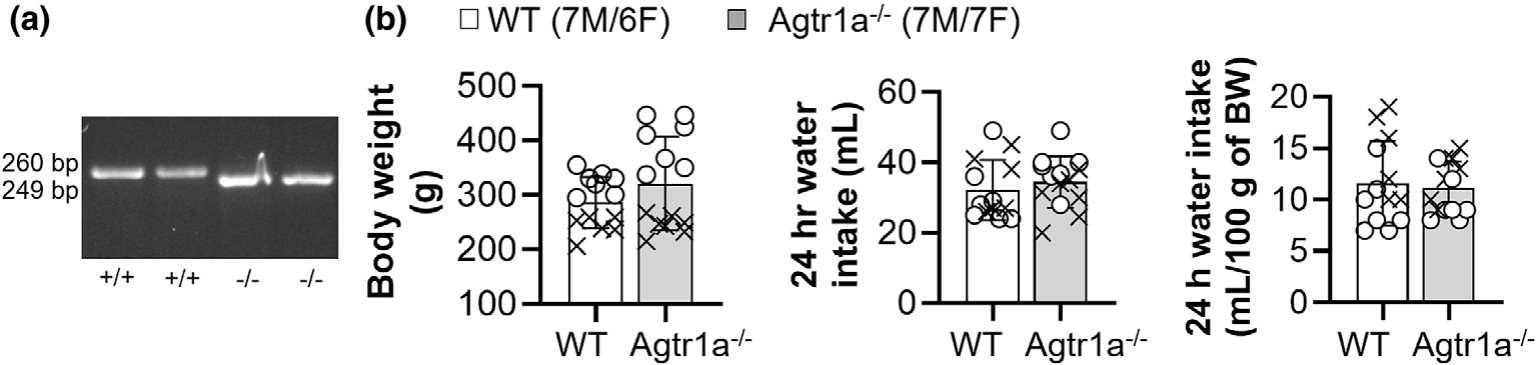
Characteristics of WT and Agtr1a^−/−^ rats. (a) Representative gel illustrating 260 and 249 bp PCR products generated from genomic DNA isolated from ear punch of WT and Agtr1a^−/−^ rats. (b) Mean ± SD and individual data points (male, Ο; female, Χ) of body weight, absolute 24‐h water intake and 24‐h water intake normalized to 100 g body weight. *p* > 0.05, unpaired *t*‐test.

Figure 1a illustrates a representative PCR gel with 260 bp and 249 bp alleles for WT and Agtr1a^−/−^ rats. There were no differences in body weight or 24‐h water intake between strains or sexes (Figure 1b). As expected, the body weights were significantly lower in female versus male rats for WT (female: 242 ± 20 vs. male: 324 ± 21, *p* < 0.0001) and Agtr1a^−/−^ (female: 245 ± 17 vs. male: 397 ± 45, *p* < 0.0001). 24‐h water intake was not different between strains (Figure 1b). When normalized to body weight, 24‐h water intake was significantly greater in female versus male in the WT group (females: 14 ± 4 vs. males: 9 ± 2 mL/100 g BW; *p* = 0.014). However, it was not statistically different for Agtr1a^−/−^ (females: 13 ± 2 vs. males: 10 ± 2 mL/100 g BW, *p* = 0.217). There were also no differences in heart weight (WT: 0.33 ± 0.05 vs. Agtr1a^−/−^: 0.31 ± 0.02 g/100 g BW) or right kidney weight (WT: 0.38 ± 0.03 vs. Agtr1a^−/−^: 0.35 ± 0.04 g/100 g BW). In contrast, the left kidney was modestly lighter in Agtr1a^−/−^ rats (*n* = 5 M/4F) compared to WT littermates (*n* = 5 M/6F) (WT: 0.36 ± 0.02 vs. Agtr1a^−/−^: 0.33 ± 0.03 g/100 g BW; *p* = 0.0146). Plasma electrolyte concentrations were measured in a subset of animals (*n* = 3–4 M/5–6F per group) during the light (or inactive) phase. There were no differences between strains in plasma [Na^+^] (WT: 141.6 ± 2.0 vs. Agtr1a^−/−^: 142.5 ± 1.8 mmol/L), plasma [Cl^−^] (WT: 106.7 ± 1.8 vs. Agtr1a^−/−^: 107.5 ± 2.8 mmol/L), and plasma [K^+^] (WT: 4.4 ± 0.4 vs. Agtr1a^−/−^: 4.3 ± 0.6 mmol/L).

**FIGURE 2:**
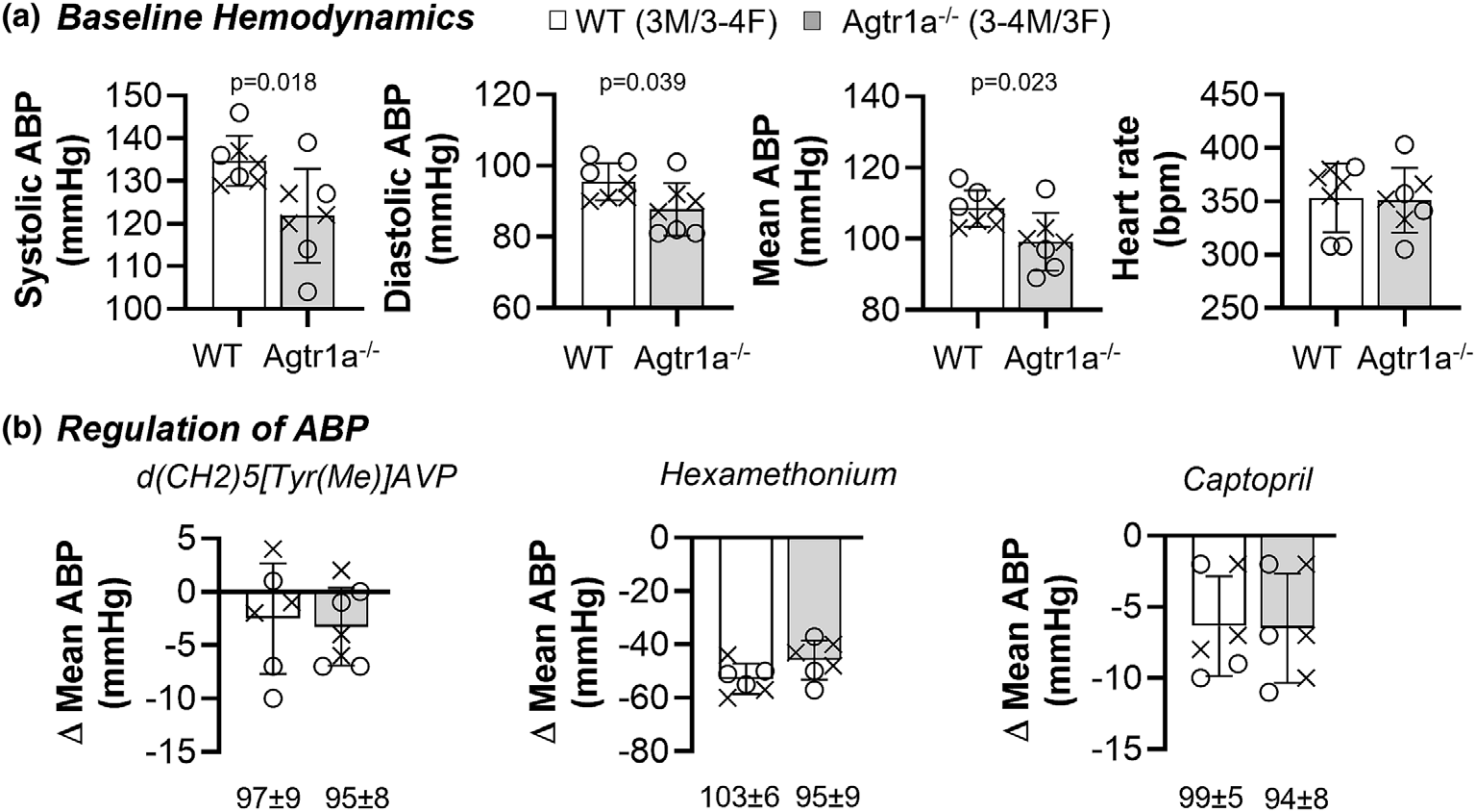
Baseline hemodynamics and regulation of ABP in WT and Agtr1a^−/−^ rats. (a) Mean ± SD and individual data points (male, Ο; female, Χ) of systolic, diastolic, and mean arterial blood pressure (ABP), and heart rate in WT and Agtr1a^−/−^ rats. Unpaired *t*‐test. (b) Mean ± SD and individual data points (male, Ο; female, Χ) of peak depressor responses after d(CH2)5[Tyr(Me)]AVP (10ug/kg, IV), hexamethonium (30 mg/kg, IV), or captopril (3 mg/kg, IV). Baseline mean ABP before each treatment is provided below each bar graph. Unpaired *t*‐test.

Baseline hemodynamics were recorded and averaged over 1‐h during the light period. Agtr1a^−/−^ rats exhibited a lower systolic ABP (WT: 135 ± 5 mmHg vs. Agtr1a^−/−^: 122 ± 11 mmHg), diastolic ABP (WT: 96 ± 5 mmHg vs. Agtr1a^−/−^: 88 ± 7 mmHg), and mean ABP (WT: 108 ± 5 mmHg vs. Agtr1a^−/−^: 99 ± 8 mmHg). In contrast, there were no differences in pulse pressure (WT: 39 ± 3 mmHg vs. Agtr1a^−/−^: 34 ± 6 mmHg) or heart rate (WT: 353 ± 32 bpm vs. Agtr1a^−/−^: 351 ± 30 bpm; Figure 2a). Next, we assessed the contribution of various pressor systems to the regulation of ABP. There were no significant differences between WT versus Agtr1a^−/−^ in the depressor responses to vasopressin 1a receptor blocker d(CH2)5[Tyr(Me)]AVP, ganglionic blocker hexamethonium, or the angiotensin converting enzyme inhibitor captopril (Figure 2b).

### 3.2 Experiment 2A: Thirst and ABP responses to IV infusion of AngII

**FIGURE 3:**
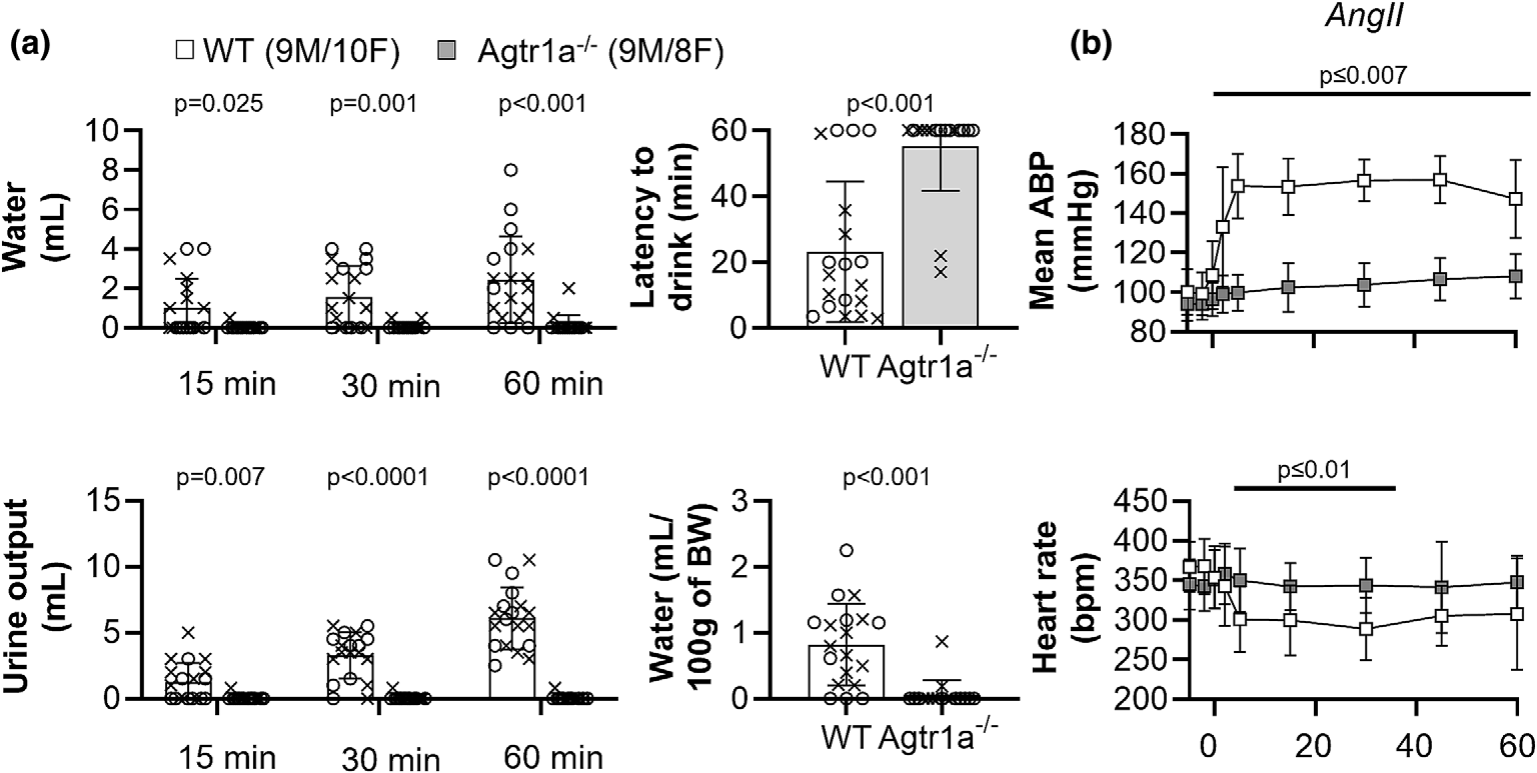
Deletion of Agtr1a abolishes thirst and cardiovascular responses induced by intravenous AngII. (a) Mean ± SD and individual data points (male, Ο; female, Χ) of water intake, urinary output, latency to drink, and water intake normalized to body weight during IV infusion of AngII (40 ng/min; 2.5 mL over 60 min) in WT and Agtr1a^−/−^ rats. Two‐way repeated‐measures ANOVA followed by Bonferroni post hoc test and unpaired *t*‐test. (b) Mean ± SD of mean ABP and heart rate during baseline and throughout AngII infusion. Two‐way repeated‐measures ANOVA followed by Bonferroni post hoc test.

To test whether the 11 bp deletion rendered the Agtr1a non‐functional, thirst and ABP responses were measured in WT and Agtr1a^−/−^ rats during IV infusion of AngII. AngII significantly increased water intake in WT but not in Agtr1a^−/−^ rats (Figure 3a). In fact, AngII stimulated thirst in a significantly greater proportion of WT versus Agtr1a^−/−^ rats (16/19 = 84% versus 2/17 = 12%, *p* < 0.001 Chi‐square) and resulted in a significantly shorter latency to drink of WT versus Agtr1a^−/−^ rats (23 ± 21 vs. 55 ± 13 min; Figure 3a). Infusion of AngII produced a significantly greater urine output (Figure 3a) and urinary electrolyte excretion (Table 1) in WT versus Agtr1a^−/−^ rats. As expected, AngII significantly increased mean ABP and decreased heart rate in WT rats but failed to increase ABP or alter heart rate in Agtr1a^−/−^ rats. No sex differences within genotypes were observed for water intake, latency to drink, urinary output and electrolyte excretion, or ABP responses. However, the bradycardic response from infusion onset to 60 min was significantly greater in males than females WT rats (data not shown).

**TABLE 1:** Urinary output and electrolyte concentrations in urine from WT and Agtr1a^−/−^ rats during IV infusion of AngII or 2M NaCl.

Treatment	Genotype	*n*	UV (mL)	Na+ (mmol)	K+ (mmol)	Cl− (mmol)
AngII	WT	9M/10F	6.1 ± 2.2	0.9 ± 0.3	0.3 ± 0.1	0.9 ± 0.3
AngII	Agtr1a^−/−^	9M/8F	0.05 ± 0.1*	0.002 ± 0.007*	0.005 ± 0.027*	0.003 ± 0.011*
2M NaCl	WT	8M/11F	11.8 ± 3.2	2.8 ± 0.7	0.4 ± 0.1	2.7 ± 0.7
2M NaCl	Agtr1a^−/−^	7M/7F	10.5 ± 4.3	2.3 ± 1.0	0.4 ± 0.1	2.3 ± 1.0

*Note*: Values are presented as mean ± SD. Agtr1a^−/−^, Agtr1a receptor knockout; UV, urinary output.

* *p* < 0.0001 versus WT, unpaired *t*‐test.

### 3.3 Experiment 2B: Hypotension‐induced thirst

**FIGURE 4:**
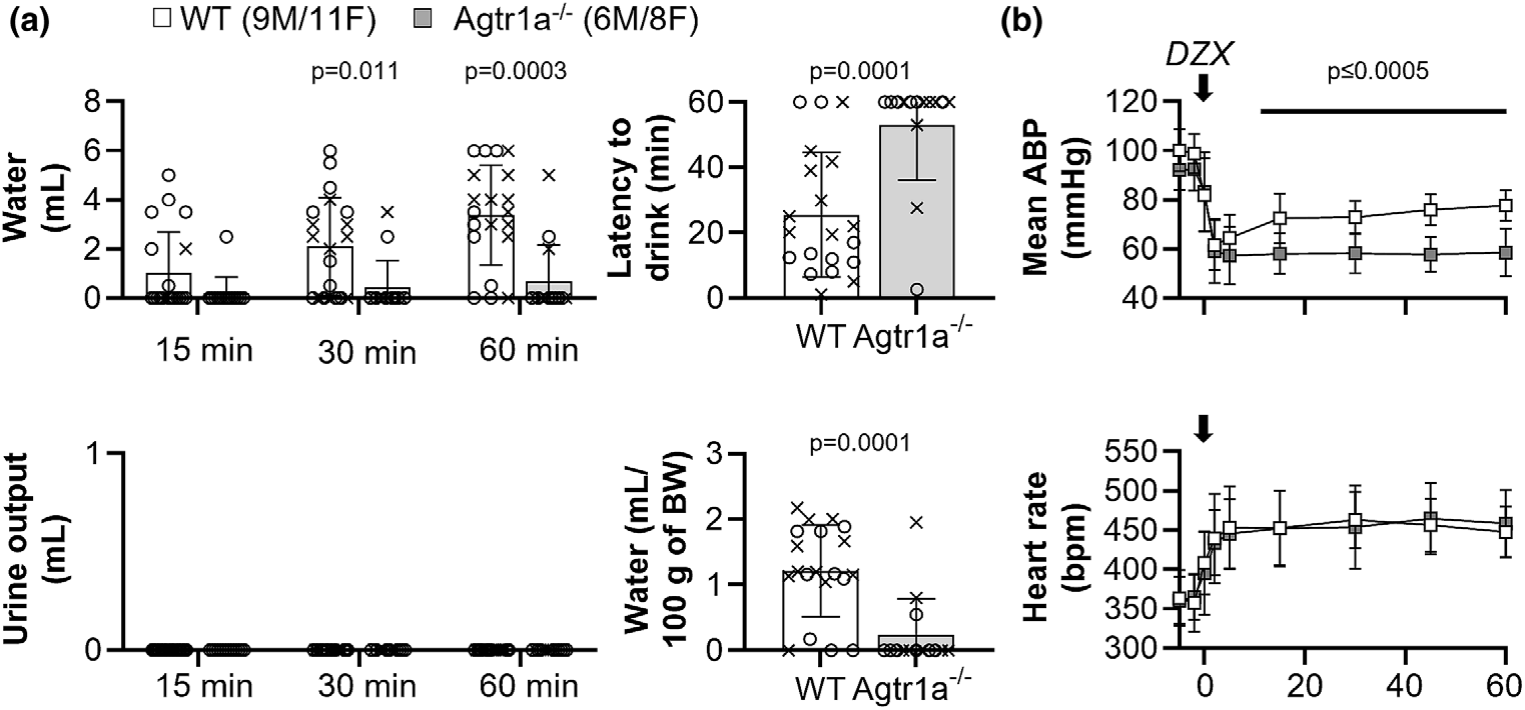
Deletion of Agtr1a impairs hypotension‐induced thirst. (a) Mean ± SD and individual data points (male, Ο; female, Χ) of water intake, urinary output, latency to drink, and 60‐min cumulative water intake normalized to body weight of WT and Agtr1a^−/−^ rats after injection of diazoxide (DZX; 25 mg/kg, IV). Two‐way repeated‐measures ANOVA followed by Bonferroni post hoc test and unpaired *t*‐test. (b) Mean ± SD of mean ABP and heart rate during baseline and 60 min after DZX. Two‐way repeated‐measures ANOVA followed by Bonferroni post hoc test.

We next examined the contribution of the Agtr1a to hypotension‐induced thirst, a response largely dependent on RAS activation. Administration of the vasodilator diazoxide readily stimulated thirst in WT but not Agtr1a^−/−^ rats (Figure 4a). Diazoxide stimulated water intake in a significantly greater proportion of WT versus Agtr1a^−/−^ rats (17/20 = 85% versus 3/17 = 21%, *p* < 0.001 Chi‐square) and resulted in a significantly shorter latency to drink of WT versus Agtr1a^−/−^ rats (25 ± 19 vs. 53 ± 16 min; Figure 4a). There were no differences in urine output between strains. Injection of diazoxide significantly decreased mean ABP in both strains (Figure 4b). Although the initial drop in mean ABP was not different between WT and Agtr1a^−/−^ rats (5 min: 64 ± 9 vs. 57 ± 11 mmHg), mean ABP remained significantly lower in Agtr1a^−/−^ versus WT rats from 15 to 60 min post injection. Injection of diazoxide significantly increased heart rate in both groups (Figure 4b). Within strains, there were no sex differences in water intake, latency to drink, or in ABP and heart rate responses at baseline or post‐infusion.

### 3.4 Experiment 2C: Hypernatremia‐induced thirst

**FIGURE 5:**
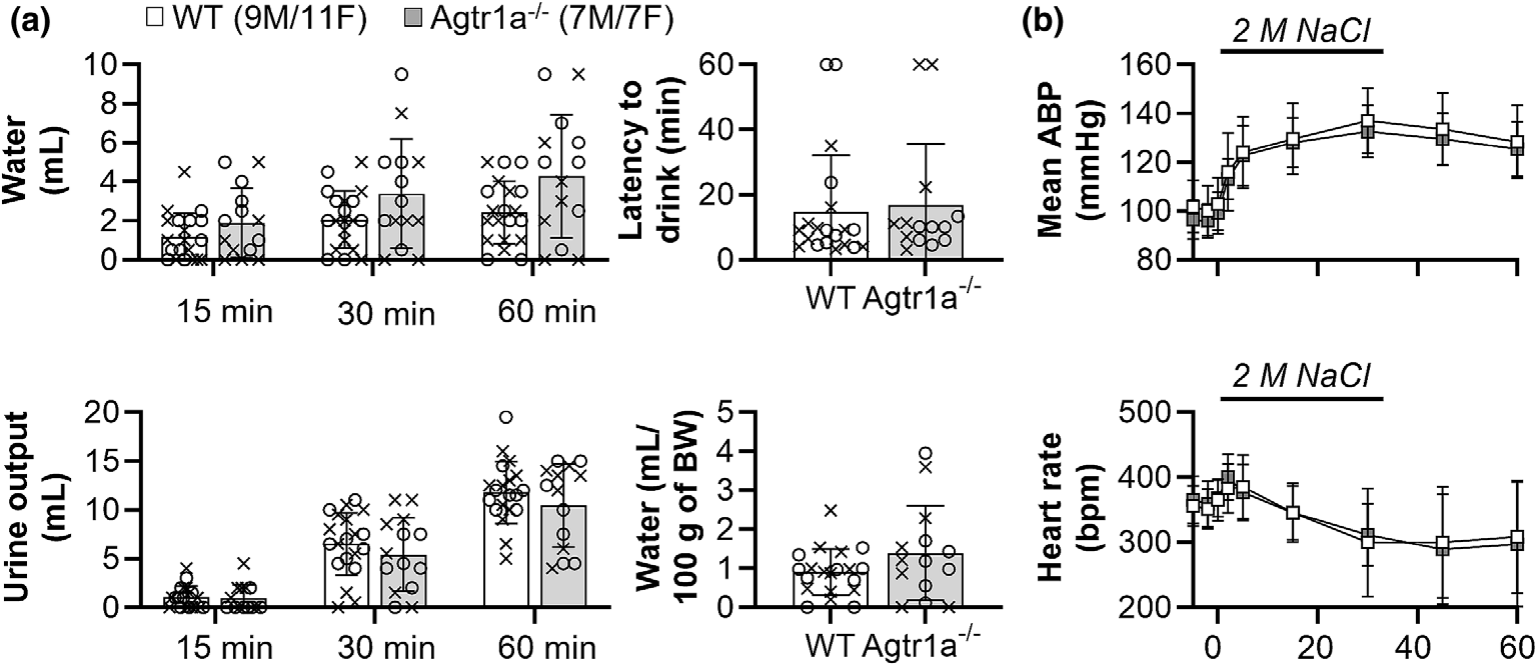
Deletion of Agtr1a did not attenuate hypernatremia‐induced thirst. (a) Mean ± SD and individual data points (male, Ο; female, Χ) of water intake, urinary output, latency to drink, and water intake normalized to body weight in response to IV infusion of hypertonic saline (2 M NaCl; 2.5 mL over 30 min) in WT and Agtr1a^−/−^ rats. *p* > 0.05, two‐way repeated measures ANOVA. *p* > 0.05, unpaired *t*‐test. (b) Mean ± SD of mean ABP and heart rate at baseline and during the infusion of hypertonic saline. *p* > 0.05, two‐way repeated‐measures ANOVA.

To test whether deletion of Agtr1a affects thirst stimulated by hypernatremia, WT and Agtr1a^−/−^ rats were infused with 2 M NaCl (2.5 mL per 30 min, IV). Acute infusion of hypertonic NaCl increased water intake in both WT and Agtr1a^−/−^ rats (Figure 5a). Water intake did not differ between strains at any time nor when normalized to BW (Figure 5a). Hypertonic NaCl stimulated water intake in a similar proportion of WT and Agtr1a^−/−^ rats (18/20 = 90% vs. 12/14 = 86%, respectively, *p* = 0.703 Chi‐Square) with no differences in latency to drink between genotypes (Figure 5a). Urinary volume (Figure 5a) and electrolyte excretion (Table 1) did not differ between groups. Infusion of hypertonic saline also increased mean ABP and decreased heart rate in both groups (Figure 5b). There were no statistical differences in either variable between genotypes. Furthermore, there were no sex differences within strain for any of the measured outcomes, with the exception of heart rate responses. Female rats exhibited lower bradycardia than males (by ~80 bpm) from 20 to 60 min after the onset of hypertonic saline infusion (data not shown).

### 3.5 Experiment 2D: Water deprivation‐induced thirst

**FIGURE 6:**
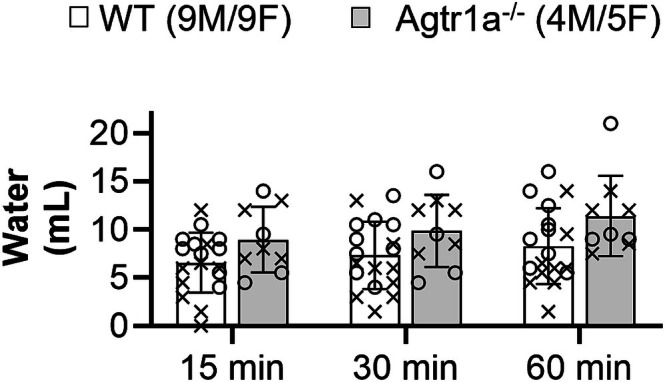
Thirst response to water deprivation is not attenuated in Agtr1a^−/−^ rats. Mean ± SD and individual data points (male, Ο; female, Χ) of water intake after 18–20 h of water deprivation in WT and Agtr1a^−/−^ rats. *p* > 0.05, two‐way repeated‐measures ANOVA.

A final thirst experiment tested whether deletion of Agtr1a altered the thirst response to 18–20 h water deprivation. Water deprivation increased water intake in both WT and Agtr1a^−/−^ rats (Figure 6). Water intake did not differ between strains at any time as well when normalized to BW (data not shown). Every WT and Agtr1a^−/−^ rat drank during the test (18/18 = 100% vs. 9/9 = 100%). Latency to drink was not measured due to the immediate onset of the response when water bottles were re‐introduced. Animals did not excrete any urine during the 60 min test. Hemodynamic changes were not assessed due to catheter patency failure in most rats. No sex differences in normalized water intake were observed within each strain (data not shown).

## 4 DISCUSSION

The present study investigated the role of Agtr1a in thirst and ABP regulation using a novel Agtr1a^−/−^ rat carrying an 11‐bp frameshift deletion generated with CRISPR‐Cas9. Baseline ABP was approximately 10 mmHg in the Agtr1a^−/−^ versus WT rats with no differences in heart rate. Thirst and pressor responses to systemic AngII were abolished in Agtr1a^−/−^ versus WT rats. Diazoxide‐induced hypotension produced a significantly smaller water intake and longer latency to drink in Agtr1a^−/−^ versus WT rats. On the other hand, thirst responses to acute hypernatremia or water deprivation were not significantly different between Agtr1a^−/−^ versus WT rats. Collectively, these findings indicate a critical role for Agtr1a in AngII and hypotension‐induced thirst.

Previous studies using intra‐arterial catheterization or telemetry report approximately 20 mmHg lower ABP in Agtr1a^−/−^ versus WT mice (Chen et al., 2005; Ito et al., 1995; Oliverio et al., 1997). Since the level ABP can influence thirst (Evered, 1992; Robinson & Evered, 1987; Stocker et al., 2001; Stocker et al., 2002), this represents a potential confounding variable to investigate the role of Agtr1a in body fluid homeostasis in mice. In the present study, deletion of Agtr1a in the rat produced only an approximate 10 mmHg lower ABP. In addition, the pressor response induced by acute IV infusion of AngII was absent, thereby confirming a non‐functional receptor. Furthermore, depressor responses to inhibition of angiotensin converting enzyme, blockade of vasopressin type 1 receptors, or ganglionic neurotransmission were not statistically different between Agtr1a^−/−^ versus WT rats. Although this experiment was likely underpowered to detect any sex differences in baseline ABP or heart rate (Figure 2), no significant sex differences in baseline ABP or heart rate were observed in larger cohorts used in subsequent experiments (Figures 3, 4, 5). Collectively, these findings establish an Agtr1a^−/−^ rat with a largely preserved baseline ABP without alterations in the regulation of baseline ABP.

AngII is a potent stimulus for thirst in the majority of species and stimulates water intake within seconds after central administration or within minutes after systemic infusion (Fitzsimons, 1998). As expected, intravenous AngII infusion in WT rats produced a rapid rise in ABP and stimulated drinking minutes later. In marked contrast, both the pressor and dipsogenic responses to AngII were absent in Agtr1a^−/−^ rats. Indeed, the majority of WT rats (84%) drank despite elevated ABP, whereas only a small fraction of Agtr1a^−/−^ rats (12%) ingested water with a relatively normal ABP. In mice, Agtr1 also mediates pressor and dipsogenic responses to intracerebroventricular AngII (Davisson et al., 2000; Li et al., 2003), but there are conflicting data regarding the receptor subtype. For example, Davisson and colleagues (Davisson et al., 2000) reported that the dipsogenic responses to central AngII were largely attenuated in Agtr1b^−/−^ mice, but the pressor responses were blunted in Agtr1a^−/−^ mice. On the other hand, Li and colleagues (Li et al., 2003) reported thirst and pressor responses to central AngII were significantly attenuated in the Agtr1a^−/−^ mice. Although we did not directly assess the role of Agtr1b, the present findings indicate Agtr1a mediates the pressor and dipsogenic actions of systemic AngII in rats.

Administration of a vasodilator decreases ABP, activates the renin‐angiotensin system, and stimulates thirst (Evered, 1990; Evered & Robinson, 1981; Stocker et al., 2000; Stricker, 1977). The hypotension‐induced thirst is mediated by the RAS as bilateral nephrectomy (Houpt & Epstein, 1971), inhibition of angiotensin converting enzyme (Evered, 1990; Evered, 1992; Evered & Robinson, 1981; Katovich et al., 1979; Stocker et al., 2000), or central blockade of Agtr1 (Fregly & Rowland, 1992) largely attenuates the associated water intake. In the present study, hypotension‐induced thirst was largely attenuated in Agtr1a^−/−^ versus WT rats. Whereas the majority of WT rats (85%) drank, only a small proportion of Agtr1a^−/−^ rats (21%) did. These differences in thirst responses were present between strains despite a significantly lower ABP in Agtr1a^−/−^ rats. The lower ABP should provide a greater activation of RAS and larger water intake (Abdelaal et al., 1976; Evered, 1990; Keeton & Campbell, 1980; Stocker et al., 2000). The lower ABP in Agtr1a^−/−^ rats after diazoxide likely reflects impaired vascular actions of AngII via Agtr1a, as prior studies have shown that ACE inhibition further reduces ABP (~15 mmHg) after diazoxide or isoproterenol in WT rats (Evered, 1990; Evered & Robinson, 1981). Collectively, these findings indicate Agtr1a plays a pivotal role in hypotension‐induced thirst.

Acute hypernatremia stimulates thirst but suppresses the systemic RAS (Abdelaal et al., 1976; Stocker et al., 2002). Bilateral nephrectomy (Fitzsimons, 1961), inhibition of angiotensin converting enzyme (Barney et al., 1980; Evered & Robinson, 1984), or central blockade of Agtr1 (Buggy & Jonklaas, 1984; Simpson et al., 1978) does not attenuate hypernatremia‐induced thirst. In the present study, deletion of Agtr1a did not attenuate thirst responses to systemic infusion of 2 M NaCl. In fact, the majority of WT (90%) and Agtr1a^−/−^ rats (86%) drank with similar latencies and ingested similar amounts of water. Renal Na^+^ excretion did not differ between the two strains. These data indicate that Agtr1a does not contribute to hypernatremia‐induced thirst (cellular dehydration) and highlights that deletion of the Agtr1a did not disrupt all forms of thirst behavior.

Water deprivation is a complex stimulus for thirst including both cellular dehydration and extracellular volume depletion. Plasma renin activity or AngII levels increase proportionately to the duration of the water deprivation (Barney et al., 1983; Mann et al., 1980; Stocker et al., 2002). However, plasma AngII levels after overnight water deprivation are slightly elevated and similar to those produced by 10 ng/kg/min AngII, which does not readily stimulate water intake in rats (Barney et al., 1983; Stocker et al., 2002). In the current study, water intake after overnight water deprivation was not different between Agtr1a^−/−^ and WT rats. It is noteworthy that central blockade of Agtr1 or inhibition of ACE does not affect thirst responses to short‐term water deprivation but does attenuate such responses as the duration of water deprivation is lengthened (Barney et al., 1983; Lee et al., 1996; Rowland & Fregly, 1993). This time‐dependency on Agtr1 is likely explained by the greater volume depletion and activation of the renin‐angiotensin system as the duration of water deprivation lengthens. The current findings indicate deletion of Agtr1a does not alter thirst in response to overnight water deprivation.

In the present study, the data strongly supports a role for Agtr1a in AngII‐ and hypotension‐induced thirst. However, we did not test its contribution to thirst induced by other forms of extracellular fluid volume depletion including hypovolemia. Hypovolemia activates the RAS (Mann et al., 1988; Stocker et al., 2001). Bilateral nephrectomy, ACE inhibition, or central AngII receptor blockade produces variable effects on PEG‐induced thirst (Buggy & Jonklaas, 1984; Evered & Robinson, 1984; Mann et al., 1988; Stricker et al., 1979). Agtr1a^−/−^ mice ingest less water than WT mice during hypovolemia (furosemide plus delayed fluid replacement), but Agtr1a^−/−^ mice exhibit a much lower baseline ABP (Matsuda et al., 2017). Hypovolemia also stimulates salt appetite (Fitzsimons, 1998; Stricker, 1981; Stricker, 1983). Although various experimental paradigms are used to induce salt appetite, RAS may also contribute, as high systemic concentrations of captopril in rats (Thunhorst & Johnson, 1994) or deletion of Agtr1a in mice (Matsuda et al., 2017) reduced salt intake stimulated by furosemide. Future experiments can address the impact of Agtr1a deletion in rats during hypovolemia‐induced thirst and salt appetite.

The current study generated a novel Agtr1a^−/−^ rat to assess the role of Agtr1 in thirst responses to a number of stimuli. It is noteworthy that angiotensin II type 2 receptors are expressed in the brain (Lenkei et al., 1997) and may contribute to elevated water intake during these experimental conditions. Rowland and Fregly (Rowland & Fregly, 1993) reported that intracerebroventricular injection of an angiotensin II type 2 receptor antagonist significantly reduced water intake to every stimulus tested including acute hypernatremia, isoproterenol, hypovolemia, water deprivation, and carbachol. Lee and colleagues (Lee et al., 1996) reported that central angiotensin II type 2 receptor antagonist attenuated water intake stimulated by water deprivation. Future studies are needed to replicate these findings and assess whether angiotensin II type 2 receptor antagonists do not produce a general disruption of ingestive behavior.

One limitation of the present study is that the Agtr1a^−/−^ rats are global knockouts. Agtr1a is highly expressed in the kidney (Crowley & Coffman, 2012; Kobori et al., 2007), and previous studies in Agtr1a‐deficient mice demonstrated hyperrenimia, increased renal renin and Agtr1b expression, altered morphology, and impaired sodium retention suggesting the development of compensatory renal adaptations (Ito et al., 1995; Oliverio et al., 1997; Sugaya et al., 1995; Zimnol et al., 2017). However, many of these changes would be expected to enhance rather than suppress thirst responses because reductions in ABP and extracellular fluid volume activate the RAAS and stimulate thirst. In contrast, Agtr1a^−/−^ rats exhibited markedly impaired thirst responses to systemic AngII and diazoxide‐induced hypotension, normal baseline water intake and plasma electrolyte concentrations, preserved hypernatremia‐induced drinking and sodium excretion. Collectively, these findings suggest preserved overall fluid homeostasis and support the interpretation that impaired drinking responses primarily reflect loss of Agtr1a‐mediated AngII signaling rather than generalized renal dysfunction or disruption of fluid‐electrolyte balance.

Future studies using tissue‐ or site‐specific deletions within the central nervous system will help to define the specific contribution of Agtr1a to thirst regulation. Radioligand binding studies have shown high Agtr1 expression in circumventricular organs (Song et al., 1992). Subsequent in situ hybridization confirmed abundant Agtr1a expression in the organum vasculosum of the lamina terminalis, subfornical organ, and median preoptic nucleus (Lenkei et al., 1998). Electrophysiological recordings have demonstrated that neurons in the organum vasculosum of the lamina terminalis (Kinsman et al., 2020) and subfornical organ respond to AngII (Felix & Schlegel, 1978; Gutman et al., 1988). Furthermore, optogenetic inhibition of OVLT excitatory neurons (Kinsman et al., 2020) or SFO lesions (Abdelaal et al., 1974) markedly reduces AngII‐induced thirst in rats. Therefore, either of these two regions or downstream circuits may be critical sites for Agtr1a signaling in thirst responses to the stimuli studied herein.

## AUTHOR CONTRIBUTIONS

**Caroline B. Ferreira:** Conceptualization; data curation; formal analysis; investigation; methodology. **Sean D. Stocker:** Conceptualization; data curation; formal analysis; funding acquisition; investigation; methodology; project administration; resources; supervision; validation.

## FUNDING INFORMATION

The research was supported by NIH Grants HL145875, HL152680, and HL163906.

## CONFLICT OF INTEREST STATEMENT

The authors have no disclosures.

## Data Availability

The data are available from the corresponding author upon reasonable request.
